# Applied Hedgehog Conservation Research

**DOI:** 10.3390/ani14060976

**Published:** 2024-03-21

**Authors:** Nigel Reeve, Anne Berger, Sophie Lund Rasmussen

**Affiliations:** 1 Independent Researcher, Woking GU21 5TR, UK; 2 Department of Evolutionary Ecology, Leibniz Institute for Zoo and Wildlife Research, Alfred-Kowalke-Straße 17, 10315 Berlin, Germany; 3 Wildlife Conservation Research Unit, Department of Biology, University of Oxford, The Recanati-Kaplan Centre, Tubney House, Abingdon Road, Abingdon OX13 5QL, UK; 4 Department of Chemistry and Bioscience, Aalborg University, Fredrik Bajers Vej 7H, DK-9220 Aalborg, Denmark; 5 Linacre College, St. Cross Road, Oxford OX1 3JA, UK

Hedgehogs (Order Eulipotyphla, Family Erinaceidae, Subfamily Erinaceinae) are familiar and popular spiny mammals, but they face many challenges in modern human-dominated environments. In this Special Issue, “Applied Hedgehog Conservation Research”, we present an anthology of articles from the journal *Animals* which help to fill some of the many gaps in our knowledge of hedgehogs and describe new approaches to their conservation. Most articles in this collection focus on the West European hedgehog (*Erinaceus europaeus*), which remains by far the most researched species to date, but studies of other hedgehog species are also included when they are relevant and informative. The articles reflect a broad and diverse spectrum of current research that is relevant to the conservation of hedgehogs in the wild and can provide insights into their behaviour, genetics, disease, and mortality, including studies of hedgehogs in human care. We gratefully acknowledge the authors who have contributed articles, the peer reviewers, and the staff of the journal, who have facilitated the production of this Special Issue. All the articles are Open Access and free to download, ensuring that the studies are available to all and can contribute significantly to evidence-based hedgehog conservation.

There is growing evidence of a serious decline in the distribution and abundance of hedgehogs in Western European countries, such as the UK [1], the Netherlands [2], Belgium [3], and Switzerland [4]. This decline of the West European hedgehog within its native range seems to be caused by a variety of factors. Different influences may be at work in urban and rural environments, but generally, we can implicate the loss, degradation, and fragmentation of suitable habitats. The isolation of small hedgehog populations may also increase the likelihood of local extinctions from stochastic events and the effects of low genetic diversity. Other negative factors include the lethal or sub-lethal effects of environmental pollutants, hazards such as road traffic accidents, a decline in the abundance of invertebrate prey, high levels of disturbance, interspecific competition, and predation, e.g., by Eurasian badgers (*Meles meles*) [2,5,6]. Despite the many negative effects of anthropogenic hazards and environmental change, hedgehogs are often found living alongside humans, especially in low-density urban and suburban areas with gardens and plenty of greenspace, where they may benefit from supplementary food sources, a reduced risk of predation, additional shelter, and a warmer microclimate [6,7,8]. Hedgehogs are typically absent from highly urbanised environments such as town centres and cities with small, highly fragmented areas of greenspace [7,9], but the benefits of an urban lifestyle can be seen in northern Scandinavian countries, where hedgehogs are principally found in association with human settlements [10], provided that an appropriate number of small forest patches in urban areas ensure suitable hibernation habitats for hedgehogs [11]. Similarly, in Qatar, a population of Ethiopian hedgehogs (*Paraechinus aethiopicus*) benefit from visiting irrigated farms and a rubbish mound [12]. However, in a contrasting example, the formerly thriving urban hedgehog population in Zurich (Switzerland) has declined in the last 25 years in both distribution (down 17.6%) and abundance (down 40.6%), and further investigations are required to establish the reasons for this [4]. 

In this Anthropocene era, the existence of almost every wildlife species depends on how well they can cope with the conditions that are created by humans. Hedgehogs are generally popular animals, but just popularity is not enough when key groups in society are disconnected from and ignorant of the natural world [13]. Citizen Science and public engagement initiatives need to be sensitive to the social context to engage and motivate people to implement conservation actions, in order to make the changes that will allow wildlife to co-exist with us. 

Urgent action is required to reverse the decline of the West European hedgehog, but there are still major gaps in our understanding of its population ecology, the underlying causes of decline, and the effectiveness of different conservation measures. Population modelling is an important strategic tool, but it requires data such as survival and reproductive rates, which are especially challenging to obtain from a secretive, nocturnal animal. If cause of death (or other additional) data can also be obtained, the models gain the power to examine further issues, such as the potential benefits of mitigations or the impact of certain causes of death (associated with certain environmental factors) on population trends [14,15]. Post-mortem age determinations of hedgehogs in Denmark [16] examined sex- and age-specific mortality rates in road-killed and other dead hedgehogs from urban and rural areas. Additional data on individual genetic heterozygosity revealed no significant association with either the age at death or the cause of death. In this case, it seems that low genetic diversity is not a principal risk factor. Moreover, hibernation is a key feature of West European hedgehogs’ life history, and for many years, it has been assumed that hibernation was a high-risk period. However, recent studies [17] show that in fact, hibernation is a period of relatively low mortality. Death from collisions with road traffic is one of the most obvious mortality factors affecting hedgehogs, and the scale of the losses suggests population-level effects [1,9]; in addition, males are more likely to be traffic victims [14,16]. The population effects of road mortality and, where implemented, the benefits of road mitigation schemes such as crossing structures and fauna tunnels need to be assessed by detailed demographic research [14]. 

As hedgehogs are elusive animals, the use of environmental DNA (eDNA) to detect their presence has considerable potential. eDNA has now been used to detect the presence of many species, including a range of terrestrial mammal species in samples of river water [18], although no hedgehogs were detected in that study. Another approach that has been successfully trialled is to use DNA analysis to look for a hedgehog-specific parasitic nematode *Crenosoma striatum* (a lungworm) in slugs—the nematode’s intermediate host [19]. Slugs are easy to collect, and this method has the potential to be used simply to confirm the presence of hedgehogs on a site, especially in habitats where fieldwork is difficult or where they are at low densities and hard to find. 

Where populations of different hedgehog species meet, much can be learned about their ecological niche characteristics, the potential for interspecific competition, and the implications for conservation management. A detailed study of cranial and jaw morphology has revealed differences between allopatric and sympatric populations of the West European hedgehog and the Northern white-breasted hedgehog (*Erinaceus roumanicus*), with a convergence in jaw shape in the sympatric animals that may be a result of feeding niche overlap and competition [20]. In the contact zone between these two European hedgehogs, wildlife rescue centres may receive both species. If releasing individuals away from their point of origin, there is a potential to create artificial mixing between their populations, irrespective of natural landscape barriers [21]. Translocations by wildlife rescue centres may also explain the unexpected gene flow that has been observed between urban subpopulations of *E. europaeus* in Berlin, although natural movements through habitat patches in the city may also be possible [22]. 

Animal rescue and rehabilitation centres aim to contribute to hedgehog welfare and conservation by restoring ailing hedgehogs to health and returning them to the wild. Bearman-Brown and Baker estimated that in 2016, at least 40,000 hedgehogs were admitted to rescue centres in Britain and the Channel Islands, of which maybe 50% were released [23]. Such figures suggest potential population-level effects. Studies that test the efficacy of rehabilitation methods [24] and provide reliable reference data for physiological variables [25] play an important part in the development of good practice for hedgehog rehabilitation. The participation of rescue and rehabilitation centres in research can be extremely valuable and can reveal much about the problems facing hedgehogs in the wild [26,27], as can studies carried out in captivity examining behaviour and hibernation [28]. Rescue centre records can provide year-on-year data on the numbers and causes of admissions and can also contribute samples to studies of, for example, infectious and non-infectious diseases; the prevalence of environmental pollution by pesticides, heavy metals, organic pollutants, and other ecotoxins [29]; plastic waste [15]; and the occurrence and characteristics of cut injuries that are presumably caused by garden tools like robotic lawn mowers [30]. Studies by Rasmussen et al. of robotic lawn mowers have shown important differences between models in the risk of injury to hedgehogs, creating the basis for specific design improvements to mitigate such risks [31,32]. Moreover, investigations of the personality and reactions of live hedgehogs towards a disarmed, approaching robotic lawn mower are also applied in the design of a standardised hedgehog safety test, eventually serving to produce and approve hedgehog-friendly robotic lawn mowers [33]. 

A range of anthropogenic influences could potentially affect hedgehogs’ behaviour. One of these is Artificial Light at Night (ALAN), which is known to disrupt the behaviour of a wide range of species. A study of hedgehogs using camera-trap videos at supplementary feeding stations in gardens found no consistent overall effect of ALAN on the feeding and general activity of hedgehogs [34]. However, a study in Berlin used GPS tracking and dataloggers and demonstrated a preference for movement in locations with lower light levels [35]. By attaching dataloggers to urban hedgehogs under different conditions, it was documented that the temporary disturbance of their habitat that was caused by a music festival had a more serious impact on hedgehog behaviour than a permanent disturbance caused by fragmentation [36]. Nevertheless, we stress the importance of further investigations on the effects of anthropogenic disturbances to the habitats before clear conclusions can be drawn. 

The popularity of hedgehogs means that many people put out food for their much-loved garden visitors. Supplementary feeding could be an important factor for maintaining urban hedgehog populations, but feeding during the winter appears, at least in some cases, to be increasing the levels and duration of activity when hedgehogs would normally be hibernating [37]. Further research is needed to evaluate the risks and benefits of supplementary feeding, facilitating evidence-based advice to the public to benefit the hedgehogs. Furthermore, contrary to the heterogeneously dispersed invertebrate prey which hedgehogs naturally forage for, supplementary feeding is usually provided in food bowls, which may artificially attract a substantial number of hedgehogs to the feeding stations. This might trigger aggression between competing hedgehogs and increase the potential risks of injury and disease transmission from close, as well as aggressive, intraspecific encounters, or contact with other competing or predatory species that are attracted to the food [38]. However, ingestion of natural prey items may also cause risks to the hedgehogs, as Williams et al. demonstrated that molluscs, commonly eaten by hedgehogs, are potential vectors of rodenticide poisons [39], providing a potential explanation for the high prevalence of rodenticides that have been detected in hedgehogs in previous studies [29,40]. 

Clearly, there is a need for further, more detailed studies to answer the many remaining questions about hedgehogs’ population ecology, habitat requirements, and behaviour, as well as the impacts of potential key causal factors in their decline. There is enough current knowledge to prescribe land management changes which are likely to benefit hedgehog populations in rural areas [41], but there is a serious lack of evidence underpinning this advice. Management interventions, or indeed any interventions aiming to benefit hedgehogs, need to be part of well-designed before/after studies to provide evidence of their effectiveness. 

We hope that the studies published in this Special Issue, “Applied Hedgehog Conservation Research”, will inspire forthcoming research and will contribute to an evidence-based optimisation of the conservation initiatives protecting this beloved species, thus preventing further population declines.
